# *Listeria monocytogenes* Brain Abscess: Controversial Issues for the Treatment—Two Cases and Literature Review

**DOI:** 10.1155/2018/6549496

**Published:** 2018-07-24

**Authors:** Beatrice Tiri, Giulia Priante, Lavinia Maria Saraca, Lucia Assunta Martella, Stefano Cappanera, Daniela Francisci

**Affiliations:** ^1^Infectious Diseases Clinic, Department of Medicine, University of Perugia, Perugia, Italy; ^2^Infectious Diseases Clinic, Department of Medicine, “S. Maria” Hospital, Terni, Italy

## Abstract

*Listeria monocytogenes* (LM) is an opportunistic pathogen, and the most common central nervous system manifestation is meningitis while listerial brain abscesses are rare. We describe 2 cases of brain abscess due to LM and a literature review. Only 73 cases were reported in the literature from 1968 to 2017. The mean age was 51.9, and the mortality rate was 27.3%. In 19% of cases, no risk factors for neurolisteriosis were identified. Blood cultures were positive in 79.5% while CSF or brain abscess biopsy material was positive in 50.8%. In 40% was started a monotherapy regimen while in 60% a combination therapy without substantial differences in mortality. Fifty-two percent underwent neurosurgery while 45.3% has been treated only with medical therapy. The mortality rates were, respectively, 13% and 38.2%. Only 25% of patients who were treated for ≤6 weeks underwent neurosurgery, while 80% of those who were treated for ≥8 weeks were operated. The mortality rates were, respectively, 12.5% and 0%, suggesting that a combined approach of surgery and prolonged medical therapy would have an impact on mortality. We believe that it is essential to carry out this review as brain abscesses are rare, and there are no definitive indications on the optimal management, type, and duration of therapy.

## 1. Introduction

*Listeria monocytogenes* (LM) is a facultative intracellular Gram-positive bacillus, widely distributed in nature and therefore found in multiple ecological sites, which can cause listeriosis, a serious foodborne bacterial infection [1]. Invasive listeriosis is classified into three forms: bacteraemia, neurolisteriosis, and maternal-neonatal infection. The incidence of listeriosis in the western hemisphere is estimated to be approximately three to six cases per 1 million population per year [2]. Epidemiological studies have identified host risk factors for bacteraemia and neurolisteriosis which include old age, innate and cellular immune deficiencies, cancer, HIV infection, cirrhosis, diabetes mellitus, alcoholism, and immunosuppressive therapies [3–6]. The most common central nervous system manifestation is meningitidis, while meningoencephalitis, rhombencephalitis, and cerebritis are less common [7]. Brain abscesses are extremely rare as they account for approximately 1–10% of CNS listerial infections and are observed in 1% of all listerial infections [8]. There are unresolved issues regarding surgical drainage of the abscess, selection of antibiotic regimen, and optimal treatment duration. We describe two cases (the first without evident immunodeficiency and the second affected by bullous pemphigoid) of brain abscess due to *Listeria monocytogenes* and discuss them by reviewing the literature on this topic.

## 2. Case Report

### 2.1. Case 1

**Figure 1:**
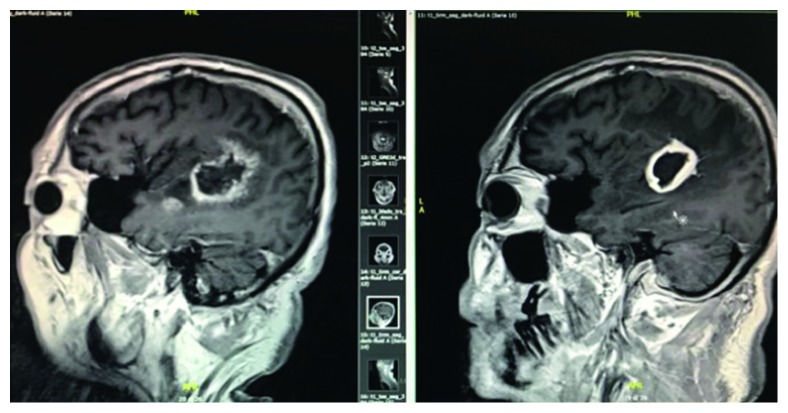
MR image showing the evolution of the ring-enhancing lesion in the left fronto-temporoparietal lobe in a brain abscess with significant perilesional edema.

A 62-year-old immunocompetent man with no significant previous medical history was hospitalized for high-grade fever, intractable hiccup, and interscapular pain. On admission, his white blood cell count was 11 × 10^9^/L (normal range 4.50–10.80 10^3^ mmc), his C-reactive protein (CRP) was elevated at 4.30 mg/dl (normal range 0.00–0.75 mg/dl), while his chest radiograph, abdomen ultrasound, and echocardiography were normal. A computed tomography (CT) scan of the brain revealed a diffuse abnormal pattern (presence of aspecific inflammatory material) with hypodense lesions located in the trigonum of lateral ventricle in an underlying condition of demyelination and gliosis, suspicious for chronic ischemic vascular disease. A broad-spectrum antibiotic therapy with vancomycin and ceftriaxone was initiated. The patient became afebrile within a few days. A neurological examination found him to be alert and oriented, and he did not have a stiff neck. However, the patient had persistent hiccups and headache. Magnetic resonance imaging (MRI) showed enhancement of both trigeminal nerves and white spot lesions on the pons, cerebral peduncle, midbrain, and thalamus. He was then transferred to the Neurology Department where a lumbar puncture was carried out. His cerebrospinal fluid (CSF) was clear, WBC count was 50 cells/*µ*l, 100% lymphocytes, normal glucose level (normal range 40–70 mg/dl), 103 mg/dl protein (normal range 15–45 mg/dl), and the CSF culture was negative. As a viral etiology was suspected, antibiotic therapy with vancomycin + ceftriaxone was discontinued and treatment with acyclovir and steroid was initiated. After 72 hours, a progressive deterioration of his clinical-neurological condition occurred: he became hyperpyretic and aphasic and Glasgow Coma Score (GCS) was 9. CT brain imaging showed the involvement of the subcortical left temporoparietal lobe, and he was then transferred to the Infectious Disease Department. Blood cultures were performed, and another lumbar puncture was carried out. A cerebrospinal fluid (CSF) analysis showed cloudy CSF with increased spinal column pressure, granulocytic pleocytosis (180 cells/*µ*l, with PMN 90%), normoglychorrachia, and 145 mg/dl spinal fluid protein. A combination antimicrobial therapy with ampicillin 3 g/6 h + gentamicin 80 mg/8 h was initiated; 72 hours later, fever and other systemic signs and symptoms disappeared resulting in complete recovery (GCS15). *Listeria monocytogenes* were isolated from the patient's blood and recognized from CSF using the molecular technique (Multiplex Real-Time PCR Meningitis/Encephalitis Filmarray bioMerieux). The patient was treated with intravenous ampicillin for 4 weeks, with combination intravenous gentamicin for the initial 2 weeks and switched to oral trimethoprim/sulfamethoxazole 160/800 mg/8 h for 1 month. An MRI was repeated after 8 weeks of antibiotic therapy due to the persistence of fluent aphasia. MR imaging showed a ring-enhancing lesion in the left fronto-temporoparietal lobe, consistent with a brain abscess with significant perilesional edema (Figure 1). Surgical excision of the lesion was performed. Molecular identification of the pus using polymerase chain reaction (PCR) identified DNA of *Listeria monocytogenes*. The patient was represcribed intravenous ampicillin + gentamicin for 4 weeks, and therapy was then switched to oral trimethoprim/sulfamethoxazole 160/800 mg/12 h for further 4 weeks. Patient's condition has improved progressively and with a complete recovery of linguistic abilities.

### 2.2. Case 2

**Figure 2:**
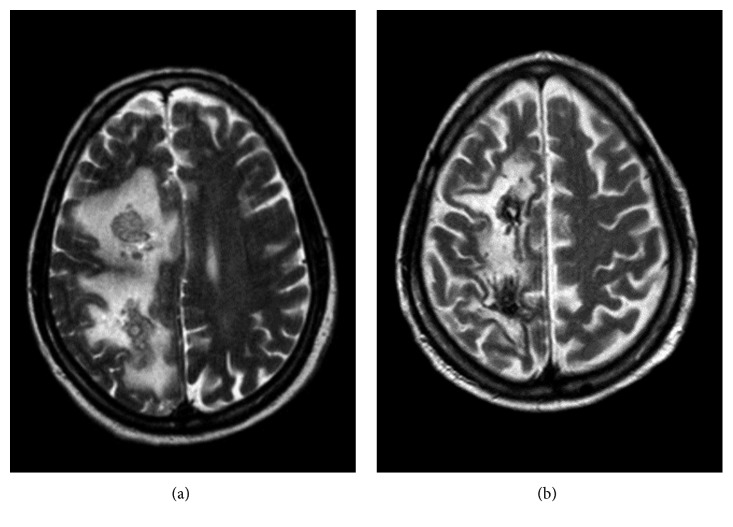
MR image showing a caudal extension of the lesion with irregular enhancement with irregular enhancement and a necrotic region (FLAIR/long TR).

A 72-year-old man with a history of bullous pemphigoid treated with a monoclonal antibody was admitted to another hospital due to a balance disorder. A neurological examination identified a left hemiplegia with no sensory deficits. An immediate CT brain scan showed a ring-enhancing cortical-subcortical lesion on the right frontal-parietal hemisphere. In view of the CT scan findings, gadolinium MRI of the brain was performed. MRI showed a caudal extension of the lesion with irregular enhancement and a necrotic region (Figure 2). Blood cultures were collected before initiating antimicrobial therapy. A few days later, his blood cultures grew *Listeria monocytogenes*. Based on organism sensitivity, intravenous therapy with ampicillin 3 g/6 h + gentamicin 80 mg/8 h + vancomycin 1 g/12 h was initiated. Steroid therapy was also administered due to the associated moderate mass effect. The patient was then transferred to our Infectious Diseases Department for further workup and management. Forty-eight hours after the initiation of target therapy, the patient was afebrile. Twenty days later, he showed progressive clinical and neurologic deterioration characterized by visual hallucinations, frontal symptoms with disinhibition, and persistent hemiplegia. An MRI brain scan showed a substantial increase in lesion size, and new lesions appeared on splenium of corpus callosum and right temporal lobe with a significant mass effect on the right lateral ventricle. Trimethoprim/sulfamethoxazole 160/800 mg/8 h was added. The patient underwent a surgical biopsy of the lesion. Molecular identification of the brain tissue using PCR identified *Listeria monocytogenes* DNA. At the follow-up appointment five weeks later, additional imaging studies were performed which showed a considerable reduction in the size and enhancement of the lesions. Ampicillin, gentamicin, and vancomycin therapy was stopped while trimethoprim/sulfamethoxazole therapy was continued. The patient's neurological condition improved. An MRI brain scan performed after 8 weeks of antibiotic therapy, showed significant improvement, with noticeable decrease in the amount of vasogenic edema. Trimethoprim/sulfamethoxazole therapy was discontinued, and the patient was discharged. A year after the listeria brain abscess diagnosis, the patient does not show any significant neurologic deficits and is able to carry out all activities of daily living.

## 3. Discussion

*Listeria monocytogenes* can invade tissues that are normally resistant to infection, such as the CNS, a gravid uterus, or a fetus. This bacterium reaches the CNS due to hematogenous spread from the gastrointestinal tract [9]. The epithelium of the choroid plexus enables LM to gain access to CNS and causes a meningitides infection. On the other hand, LM may reach the brain parenchyma via the cerebral capillary endothelium, a single layer of brain microvascular endothelial cells characterized by tight junctions. It has been reported that LM-infected macrophages may pass through endothelial cells via the middle cerebral artery resulting in cerebritis which leads to brain abscess formation [10–13].

Furthermore, LM can use a peripheral intraneural route to invade the CNS. A recent animal study suggests that once the bacteria have gained access to the CNS via the peripheral nervous system, the infection can spread along the axons, producing additional lesions by traveling within the axons of the trigeminal nerve [14–16]. According to Bojanowski et al., once inside the CNS, the bacterium may travel along the white fiber tracts of the brain, resulting in a distinct anatomical imaging thus enabling early diagnosis [17]. The spreading of multiple listeria brain abscess within the cerebral nervous system through the intrassonal pathway justified their specific pattern and why they have more detrimental effects than bacterial brain abscess. In our case 1, MRI shows that the spreading follows the arcuate fasciculus. In case 2, the caudal extension of the lesions may also suggest that the lesion follows the projection fiber tracts.

Brain abscesses are extremely rare, accounting for approximately 1–10% of CNS listerial infections. These abscesses are generally located in the subcortical grey matter, especially in the thalamus and basal ganglia [18, 19]. Protection against LM is predominantly cell-mediated. Individuals with impaired cell-mediated immunity are at risk of developing listerial infections [20].

To the best of our knowledge, only 73 cases of brain abscess caused by *L. monocytogenes* were reported in the literature between 1968 and 2017. We report further two cases (Table 1) [1, 13, 17, 21–23].

Forty-eight of these patients were male (64%). The mean age of the patients was 51.9, and median age was 55 years (range 0–87 years). Fifty-nine out of 73 had one or more risk factors described in the literature for the development of neurolisteriosis (81%), 15/75 had no risk factors (19%), and in 1 case, nothing was specified. The mortality rate was 27.3%.

Blood cultures were reported for 63 cases: 50/63 were positive (79.5%).

*L. monocytogenes* was isolated from the CSF or brain abscesses in 31/61 patients (50.8%).

The therapeutic regimen was reported for 67/75 cases, while it is unknown in 8/75.

Twenty-seven out of 67 patients received a monotherapy regimen (40%), while a combination therapy was prescribed for 40/67 (60%) cases: a two-drug therapy was prescribed in 31 cases (50.8%) and a three-drug therapy was administered in 9 cases (14.7%).

The mortality rate in the monotherapy regimen group was 18.5% (five patients out of 27) while the group that received combination therapy showed a 20% mortality rate (eight patients out of 40). Fifty-nine out of 67 patients received a beta-lactam regimen, while 8/59 received a free beta-lactam regimen.

Considering the substantial numerical difference of the two samples, these are not comparable.

Ampicillin was the most commonly prescribed antibiotic as it was administered to 49 patients: in 21 patients, it was prescribed as monotherapy; in 23 cases, it was administered in combination with gentamicin; in 3 cases, it was administered in combination with trimethoprim/sulfamethoxazole while in 2 cases, it was administered in combination with other drugs such as vancomycin or macrolides.

There are currently no guidelines for brain abscess management. Starting from the 2010 consensus on the management and treatment of brain abscesses, we reviewed our case series [24].

Thirty-nine out of 75 patients underwent neurosurgery (52%). Four out of 31 died (13%). Thirty-four patients out of 75 (45.3%) had only been treated with medical therapy. Of these, 15/34 died (38.2%). In 2 cases, no data have been reported.

Therefore, in our case series, taking into account all of the possible bias, mortality would appear to be significantly higher in the group of patients treated exclusively with medical therapy.

In our opinion, this is a very interesting finding which requires further investigation.

However, as yet, there is no evidence concerning the appropriate duration of therapy for those patients who underwent neurosurgery.

According to a recent consensus study, antimicrobial treatment for brain abscesses should generally last 6–8 weeks and treatment for those undergoing neurosurgery should last 4–6 weeks [24].

From our literature review, the duration of therapy was known in 36/75 patients. Sixteen out of 36 received less than or equal to 6 weeks while 20/36 patients were treated for 8 weeks or more. Of the group of patients who received ≤6 weeks of therapy, 4/16 (25%) underwent neurosurgery, while of those belonging to the group who received ≥8 weeks, 16/20 (80%) underwent neurosurgery.

A 12.5% mortality rate was observed for the first group while 0% died in the second group, thus suggesting that a combination of surgery and prolonged medical therapy has a positive impact on mortality.

We believe that it is essential to carry out this review as brain abscesses are rare, and there are no definitive guidelines on the optimal management, type, and duration of therapy. LM infection should also be suspected in immunocompetent patients, and new molecular biology techniques play key roles in the early diagnosis of this rare pathology.

## 4. Conclusions

In our literature review, we found that listeria brain abscess is not related to advanced age and that it is related to high mortality (27.3%).

Diagnosis should not be suspected only in immunocompromised patients as it was found in 20% of patients who had no risk factor.

Blood cultures were positive in more than 80% of cases. Most patients received a beta-lactam regimen, and mortality appears to be lower in patients treated with combination regimens.

This result looks certainly very interesting and should be explored with dedicated studies (i.e., sharp difference in mortality between the group undergoing neurosurgery and the group that only received medical therapy). Furthermore, the specific pattern of brain diffusion, reported and highlighted in our two clinical cases, should be considered when this diagnosis is hypothesized.

## Figures and Tables

**Table 1:** Seventy-three cases of brain abscess caused by *Listeria monocytogenes* reported in the literature between 1968 and 2017 (we described two other cases).

N.	Age/sex	Underlying diseases	Blood	CSF/brain abscess	Surgery/type	Antibiotic	Duration of therapy	Outcome	References
1	70/M	Myasthenia gravis in immunosuppressive TP	+	−	ND	Ampicillin + gentamicin; trimethoprim/sulfamethoxazole	6 weeks ampicillin + gentamicin for 10 days trimethoprim/ sulfamethoxazole	Survived	Chalouhi et al., 2013
2	57/F	Cirrhosis; DM	+	+	Biopsy	Ampicillin + gentamicin	NR	Died	Matera et al., 2012
3	60/M	DM; rheumatoid arthritis methotrexate	−	+	ND	(a) Amoxicillin + trimethoprim/sulfamethoxazole (b) Trimethoprim/sulfamethoxazole (c) Linezolid (d) Amoxicillin	(a) 17 days (b) 20 days (c) 33 days (d) NR	Survived	Coste et al., 2012
4	52/M	OLT in HCC secondary to hepatitis C and alcoholic cirrhosis; cyclosporine	+	+	Craniotomy with resection of the lesion	Ampicillin + gentamicin + penicillin G	3 weeks (gentamicin only for 2 weeks)	Survived	Choudhury et al., 2013
5	56/F	Primary biliary cirrhosis; OLT; tacrolimus, azathioprine, prednisone	+	+	Biopsy	Ampicillin + gentamicin	8 weeks (gentamicin only for 2 weeks)	Survived	Tseng et al., 2013
6	42/M	None	+	+	Biopsy and drainage	Ampicillin + gentamicin + meropenem	NR	Survived	Beynon et al., 2013
7	47/F	SLE; mycophenolate	−	+	ND	Ampicillin	6 weeks	Survived	Horta-Baas et al., 2013
8	16/F	SLE; mycophenolate	+	−	External ventricles device	Trimethoprim/sulfamethoxazole + ampicillin + meropenem	13 weeks trimethoprim/sulfamethoxazole; ampicillin for 4 weeks; meropenem for 5 weeks (total of 22 weeks)	Survived	Perini et al., 2014
9	81/M	Myelodys plastic syndrome; basal cell skin carcinoma, prostate cancer treated	+	+	Craniotomy with resection of the lesion	Ampicillin	NR	Survived	West et al., 2015
10	52/F	DM, hypothyroidism, prednisolone, azathioprine	+	−	Biopsy	Ampicillin + gentamicin	6 weeks	Survived	Al-HarabI et al., 2015
11	81/F	DM	NR	+	Biopsy	Ampicillin	8 weeks	Survived	Dejesus-Alvelo et al., 2015
12	74/F	DM	+	NR	NR	Vancomycin + ampicillin + ceftriaxone	NR	Survived	Bojanowski et al. [17]
13	32/F	LAC	+	NR	NR	Ampicillin + trimethoprim/sulfamethoxazole + linezolid	8 weeks; linezolid for 10 days	Survived	Fervienza et al., 2016
14	72/M	None	−	+	ND	Ampicillin	NR	Survived	Mano et al., 2017
15	52/M	Inflammatory myositis treated with prednisolone and azathioprine	+	−	ND	Ampicillin	6 weeks	Survived	Onder et al., 2016
16	70/M	Alcoholism	+	+	ND	Ampicillin + gentamicin + vancomycin	3–6 weeks	Died	Cone et al. [13]
17	56/M	AIDS	+	−	ND	Ampicillin + gentamicin	Article not available	Survived	Patey et al., 1989
18	49/M	Rheumatic fever, alcoholism, DM	+	−	ND	Penicillin G + streptomycin + tetracycline	NR	Died	Buchner and Schneierson, 1968
19	64/M	DM, aortic valve replacement	+	−	ND	Ampicillin + gentamicin	4 weeks + ampicillin for 2 weeks	Survived	Soto and Sliman, 1992
20	71/M	DM, rheumatic heart disease	+	−	ND	Ampicillin + gentamicin	NR	Died	Eckburg et al. [22]
21	56/M	AIDS	+	−	ND	Ampicillin + gentamicin	Article not available	Survived	Patey et al., 1989
22	70/F	Cirrhosis, DM, heart failure	+	ND	ND	Ampicillin + trimethoprim/sulfamethoxazole	Article not available	Died	Sivalinga et al., 1992
23	25/F	Ulcerative colitis	NR	NR	ND	NR	NR	Died	Larsson and Linell, 1979
24	87/M	None	+	+	ND	Penicillin G + chloramphenicol	NR	Died	Spilkin et al., 1968
25	63/M	None	+	−	ND	Ampicillin	NR	Died	Kennard et al., 1979
26	24/M	None	+	−	ND	Ampicillin + gentamicin	6 weeks, gentamicin only for 10 days	Survived	Smiatacz et al., 2006
27	53/F	None	+	−	ND	Minocycline, gentamicin	2 weeks	Survived	Mrowka et al., 2002
28	63/F	None	−	−	ND	NR	NR	Died	Brun-Buisson et al., 1985
29	43/F	None	−	−	ND	Ampicillin	NR	Died	Brun-Buisson et al., 1985
30	39/M	None	NR	−	ND	NR	NR	Died	Kwantes and Isaac, 1971
31	54/F	None	NR	NR	ND	NR	NR	Died	Larsson and Linell, 1979
32	1+1/4/M	None	NR	NR	ND	Amoxicillin	Article not available	Survived	Mancini et al., 1990
33	70/M	NONE	−	+	Craniectomy and open biopsy	Ampicillin	NR	Survived	Salgado et al., 1996
34	53/M	Cirrhosis, seizure	+	−	+	Penicillin G + erythtomycin	Article not available	Survived	Halkin et al., 1971
35	85/M	DM	+	−	+	Ampicillin	Article not available	Died	Brown et al., 1991
36	43/M	OSAS, alcoholism	+	−	+	NR	Article not available	Survived	Douen and Bourque, 1997
37	0/M	Pronatis	+	−	+	Ampicillin + gentamicin	Article not available	Survived	Banerji and Noya, 1999
38	63/M	MM	+	−	Biopsy	(a) Ampicillin	(a) 5 weeks	Survived	Leiti et al. [20]
						(b) Linezolid + rifampin	(b) 15 weeks		
39	61/M	DM	NR	NR	Biopsy	Trimethoprim/sulfamethoxazole + chloramphenicol	3 weeks, trimethoprim/sulfamethoxazole alone for 20 weeks	Survived	Sjostrom et al., 1995
40	60/M	HIV	NR	+	Craniotomy and intraoperative cultures	Penicillin G + chloramphenicol	NR	Died	Harris et al., 1989
41	68/M	Leukemia	NR	NR	ND	Chloramphenicol	NR	Died	Larsson et al., 1978
42	NR/M	None	NR	NR	ND	NR	NR	Died	Pollock et al., 1984
43	2/M	NR	NR	+	Craniotomy with resection of the lesion	NR	NR	Survived	Umenai et al., 1978
44	49/M	Renal transplant	+	+	ND	Chloramphenicol	NR	Died	Crocker and Leicester, 1976
45	16/M	ALL	+	+	ND	Penicillin G + chloramphenicol	NR	Survived	Dykes et al., 1979
46	20/M	ALL	+	+	ND	Ampicillin + chloramphenicol + erythromycin, gentamicin	8 weeks	Survived	Hutchinson and Heyn, 1983
47	6/F	ALL	+	+	ND	Ampicillin, vancomycin, netilmicin	NR	Survived	Viscoli et al., 1991
48	46/F	Ulcerative colitis	+	+	ND	Ampicillin, gentamicin	8 weeks, 4 weeks	Survived	Soares-Fernandes et al., 2008
49	58/F	SLE	+	−	ND	Penicillin G + tobramycin	ARTICLE NOT AVAILABLE	Survived	Takano et al., 1999
50	58/F	Immunoblastic lymphadenopathy	+	−	ND	Ampicillin	8 weeks	Survived	Maezawa et al. [21]
51	65/M	DM	+	NR	ND	Ampicillin + gentamicin	4 weeks	Died	Wu et al., 2010
52	19/M	Juvenile rheumatoid arthritis, tetralogy of Fallot	NR	+	ND	Vancomycin + ampicillin	Article not available	Survived	Turner et al., 1995
53	55/M	Renal transplant	+	−	+	Ampicillin	Article not available	Survived	Lechtenberg et al., 1979
54	45/M	Renal transplant	+	−	Craniotomy and drainage	Ampicillin	10 weeks	Survived	Stam et al., 1982
55	60/F	Rheumatoid arthritis	+	−	Biopsy	Ampicillin, amoxicillin	8 weeks, 24 months	Survived	Updike et al., 1990
56	66/F	AML, Crohn's disease	+	+	Biopsy	Ampicillin	4 weeks	Survived	Eckburg et al. [22]
57	47/M	AIDS	+	−	Craniotomy	Ampicillin, gentamicin, vancomycin	NR	Died	Cone et al. [13]
58	54/F	Sarcoidosis	+	−	Biopsy	Ampicillin + gentamicin	NR	Died	Ackermann et al., 2001
59	23/F	ITP	+	−	Drainage of the abscess	Trimethoprim/sulfamethoxazole	12 months	Survived	Treebupachatsaul et al., 2006
60	58/M	MM	+	−	Craniotomy and drainage	(a) Trimethoprim/sulfamethoxazole + gentamicin (b) Trimethoprim/sulfamethoxazole	(a) 12 weeks (gentamicin only 2 weeks) (b) 5 months	Survived	Al-Khatti and Al- Tawfiq, 2010
	
61	55/M	Glioblastoma multiforme	−	+	Biopsy	Amoxicillin + gentamicin	12 weeks	Survived	Ganiere et al., 2006
62	51/M	Cardiac transplant	+	+	Stereotactic brain aspiration	Ampicillin + gentamicin	6 weeks, gentamicin only 2 weeks	Survived	Eckburg et al. [22]
63	37/M	Cardiac transplant	+	+	Craniotomy with resection of the lesion	penicillin G	8 weeks	NR	Eckburg et al. [22]
64	56/F	Primary biliary cirrhosis	+	+	Biopsy	Ampicillin + gentamicin	6 weeks gentamicin only 2 weeks	Survived	Cone et al. [13]
65	50/M	Sarcoidosis	−	+	Craniotomy	Trimethoprim/sulfamethoxazole	Article not available	Survived	Poropatich and Phillips, 1992
66	51/F	Crohn's disease	−	+	Biopsy	Ampicillin + gentamicin	12 weeks (gentamicin not reported)	Survived	Stefanovich et al., 2010
67	50/M	Cardiac transplant, DM	−	+	Biopsy and aspiration	Ampicillin + gentamicin	18 weeks of ampicillin; 14 weeks gentamicin	Survived	Eckburg et al. [22]
68	75/M	None	−	ND	+	Ampicillin + gentamicin	Article not available	Survived	Mylonakis et al., 1998
69	77/M	CLL	−	NR	+	Chloramphenicol	Article not available	NR	Cleveland and Gelfand, 1993
70	58/M	CLL	−	+	Biopsy	Ampicillin + gentamicin	6 weeks	Survived	Dee and Lorber, 1986
71	Child	ALL	NR	NR	+	NR	Article not available	Survived	Antunes et al., 1998
72	68/F	Breast cancer	+	ND	Biopsy	Ampicillin, amoxicillin	10 weeks, 24 weeks	Survived	Limmahakhun and Chayakulkeeree [1]
73	47/F	Evans syndrome, SLE, DM	+	−	ND	Ampicillin, amoxicillin	6 weeks, NR	Survived	Limmahakhun and Chayakulkeeree [1]
Case 1	62/M	None	+	+	Craniotomy with resection of the lesion	(a) Ampicillin + gentamicin (b) Trimethoprim/sulfamethoxazole	(a) 8 weeks (gentamicin only 4 weeks) (b) 8 weeks	Survived	
	
Case 2	72/M	Bullous pemphigoid	+	+	Biopsy	(a) Ampicillin + gentamicin + trimethoprim/sulfamethoxazole (b) Trimethoprim/sulfamethoxazole	(a) 5 weeks (b) 3 weeks	Survived	
	

## References

[1] Limmahakhun S., Chayakulkeeree M. (2013). *Listeria monocytogenes* brain abscess: two cases and review of the literature. *Southeast Asian Journal of Tropical Medicine and Public Health*.

[2] Pagliano P., Ascione T., Boccia G., De Caro F., Esposito S. (2016). *Listeria monocytogenes* meningitis in the elderly: epidemiological, clinical and therapeutic findings. *Le Infezioni in Medicina*.

[3] De Noordhout C. M., Devleesschauwer B., Angulo F. J. (2014). The global burden of listeriosis: a systematic review and meta-analysis. *The Lancet Infectious Diseases*.

[4] Pagliano P., Arslan F., Ascione T. (2017). Epidemiology and treatment of the commonest form of listeriosis: meningitis and bacteraemia. *Le Infezioni in Medicina*.

[5] Charlier C., Perrodeau É., Leclercq A. (2017). Clinical features and prognostic factors of listeriosis: the MONALISA national prospective cohort study. *The Lancet Infectious Diseases*.

[6] Vázquez-Boland J. A., Kuhn M., Berche P. (2001). Listeria pathogenesis and molecular virulence determinants. *Clinical Microbiology Reviews*.

[7] Morosi S., Francisci D., Baldelli F. (2006). A case of rhombencephalitis caused by *Listeria monocytogenes* successfully treated with linezolid. *Journal of Infection*.

[8] Lorber B. (1997). Listeriosis. *Clinical Infectious Diseases*.

[9] Vázquez-Boland J. A., Krypotou E., Scortti M. (2017). Listeria placental infection. *mBio*.

[10] Disson O., Lecuit M. (2012). Targeting of the central nervous system by *Listeria monocytogenes*. *Virulence*.

[11] Drevets D. A., Bronze M. S. (2008). *Listeria monocytogenes*: epidemiology, human disease, and mechanisms of brain invasion. *FEMS Immunology & Medical Microbiology*.

[12] Schlüter D., Chahoud S., Lassmann H., Schumann A., Hof H., Deckert-Schlüter M. (1996). Intracerebral targets and immunomodulation of murine *Listeria monocytogenes* meningoencephalitis. *Journal of Neuropathology and Experimental Neurology*.

[13] Cone L. A., Leung M. M., Byrd R. G., Annunziata G. M., Lam R. Y., Herman B. K. (2003). Multiple cerebral abscesses because of *Listeria monocytogenes*: three case reports and a literature review of supratentorial listerial brain abscess(es). *Surgical Neurology*.

[14] Dons L., Weclewicz K., Jin Y., Bindseil E., Olsen J. E., Kristensson K. (1999). Rat dorsal root ganglia neurons as a model for *Listeria monocytogenes* infections in culture. *Medical Microbiology and Immunology*.

[15] Guldimann C., Lejeune B., Hofer S. (2012). Ruminant organotypic brain-slice cultures as a model for the investigation of CNS listeriosis. *International Journal of Experimental Pathology*.

[16] Oevermann A., Di Palma S., Doherr M. G., Abril C., Zurbriggen A., Vandevelde M. (2010). Neuropathogenesis of naturally occurring encephalitis caused by *Listeria monocytogenes* in ruminants. *Brain Pathology*.

[17] Bojanowski M. W., Seizeur R., Effendi K., Bourgouin P., Magro E., Letourneau-Guillon L. (2015). Spreading of multiple *Listeria monocytogenes* abscesses via central nervous system fiber tracts: case report. *Journal of Neurosurgery*.

[18] Bartt R. (2000). Listeria and atypical presentations of Listeria in the central nervous system. *Seminars in Neurology*.

[19] Matano S., Satoh S., Harada Y., Nagata H., Sugimoto T. (2010). Antibiotic treatment for bacterial meningitis caused by *Listeria monocytogenes* in a patient with multiple myeloma. *Journal of Infection and Chemotherapy*.

[20] Leiti O., Gross J. W., Tuazon C. U. (2005). Treatment of brain abscess caused by *Listeria monocytogenes* in a patient with allergy to penicillin and trimethoprim-sulfamethoxazole. *Clinical Infectious Diseases*.

[21] Maezawa Y., Hirasawa A., Abe T. (2002). Successful treatment of listerial brain abscess: a case report and literature review. *Internal Medicine*.

[22] Eckburg P. B., Montoya J. G., Vosti K. L. (2001). Brain abscess due to *Listeria monocytogenes*: five cases and a review of the literature. *Medicine*.

[23] Samra Y., Hertz M., Altmann G. (1984). Adult listeriosis–a review of 18 cases. *Postgraduate Medical Journal*.

[24] Arlotti M., Grossi P., Pea F. (2010). Consensus document on controversial issues for the treatment of infections of the central nervous system: bacterial brain abscesses. *International Journal of Infectious Diseases*.

